# Stearic acid induces proinflammatory cytokine production partly through activation of lactate-HIF1α pathway in chondrocytes

**DOI:** 10.1038/srep13092

**Published:** 2015-08-14

**Authors:** Hongming Miao, Liang Chen, Lijun Hao, Xuan Zhang, Yujuan Chen, Zhihua Ruan, Houjie Liang

**Affiliations:** 1 Department of Oncology, Southwest Hospital, Third Military Medical University, Chongqing 400038, China; 2 Department of Orthopedics, Daping Hospital, Third Military Medical University, Chongqing 400042, China

## Abstract

The biomechanics stress and chronic inflammation in obesity are causally linked to osteoarthritis. However, the metabolic factors mediating obesity-related osteoarthritis are still obscure. Here we scanned and identified at least two elevated metabolites (stearic acid and lactate) from the plasma of diet-induced obese mice. We found that stearic acid potentiated LDH-a-dependent production of lactate, which further stabilized HIF1α protein and increased VEGF and proinflammatory cytokine expression in primary mouse chondrocytes. Treatment with LDH-a and HIF1α inhibitors notably attenuated stearic acid-or high fat diet-stimulated proinflammatory cytokine production *in vitro* and *in vivo*. Furthermore, positive correlation of plasma lactate, cartilage HIF1α and cytokine levels with the body mass index was observed in subjects with osteoarthritis. In conclusion, saturated free fatty acid induced proinflammatory cytokine production partly through activation of a novel lactate-HIF1α pathway in chondrocytes. Our findings hold promise of developing novel clinical strategies for the management of obesity-related diseases such as osteoarthritis.

Osteoarthritis (OA), characterized by irreversible destruction of the joint cartilage, is a most common rheumatic disease^1^. Overnutrition-induced obesity is suggested to be a crucial contributor of the onset and progression of OA^2^, with the molecular mechanism being largely unknown. Previous studies revealed that two aspects might be the initiators of obesity-related OA. One is the biomechanics stress in obesity^3^, and the other is obesity-induced low grade inflammation^4^, characterized by activation of proinflammatory signaling pathway (TLR4, NF-κB, JNK, etc) and elevation of proinflammatory cytokines (IL-6, IL-1β, TNF-α, etc) in multiple organs and tissues^5^,^6^,^7^,^8^.

Actually, besides some cytokines and hormones (VEGF^9^, leptin^10^, etc), some increased metabolites are also potent mediators of chronic inflammation in obesity. Saturated free fatty acids (FFAs) are well-documented stimulators of toll-like receptor 4 (TLR4) in macrophages, and contribute to obesity-related chronic inflammation and insulin resistance^5^,^6^,^11^. Cholesterols play important roles in the recruitment of macrophages and onset of inflammation in adipose tissues^12^. Our previous studies revealed that deficiency of comparative gene identification-58 in macrophages induced lipid accumulation and systemic inflammation^6^,^7^.

Obesity-induced metabolites regulate proinflammatory cytokine production through a large sum of transcription factors, like NF-κB^13^, forkhead box-containing protein O subfamily-1^7^, hypoxia-inducible factors (HIFs)^14^,^15^, etc. HIFs are composed of two dimeric subunits: an oxygen-sensitive α subunit (HIF1α or HIF2α) and a ubiquitously and constitutively expressed β subunit (HIF1β)^16^. Under normal conditions, HIF1α is hydroxylated by prolylhydroxylases, ubiquitinated by the ubiquitin ligase and targeted for proteolytic degradation via the proteasomal pathway. The hydroxylation step is inhibited under hypoxic conditions, resulting in stabilization of HIF1α^16^.

Recently, HIF1α was reported to be a proinflammatory transcription factor in adipose tissue in obesity^14^,^17^. The obesity state-induced hypoxia was suggested to be a major stimulator of HIF1α expression and activity^14^. Excessive oxidation of FFAs in obesity consumed too much oxygen, leading to a hypoxia circumstance. Consequently, lactic acid/lactate will be abundantly produced via increased glycolysis. However, the relationships between the metabolic factors (FFAs, lactate, etc), HIF1α and inflammation are still not fully understood. Here in this study, we demonstrate that the high fat diet (HFD) and stearic acid induce lactate production in mice and chondrocytes, respectively. The increased lactate stabilizes HIF1α, partly mediating FFAs-stimulated proinflammatory cytokine production in chondrocytes. Our findings revealed a novel molecular mechanism linking obesity to OA.

## Results

### Increased production of proinflammatory cytokines and VEGF in the plasma and chondrocytes in the HFD-feeding mice

**Figure 1:**
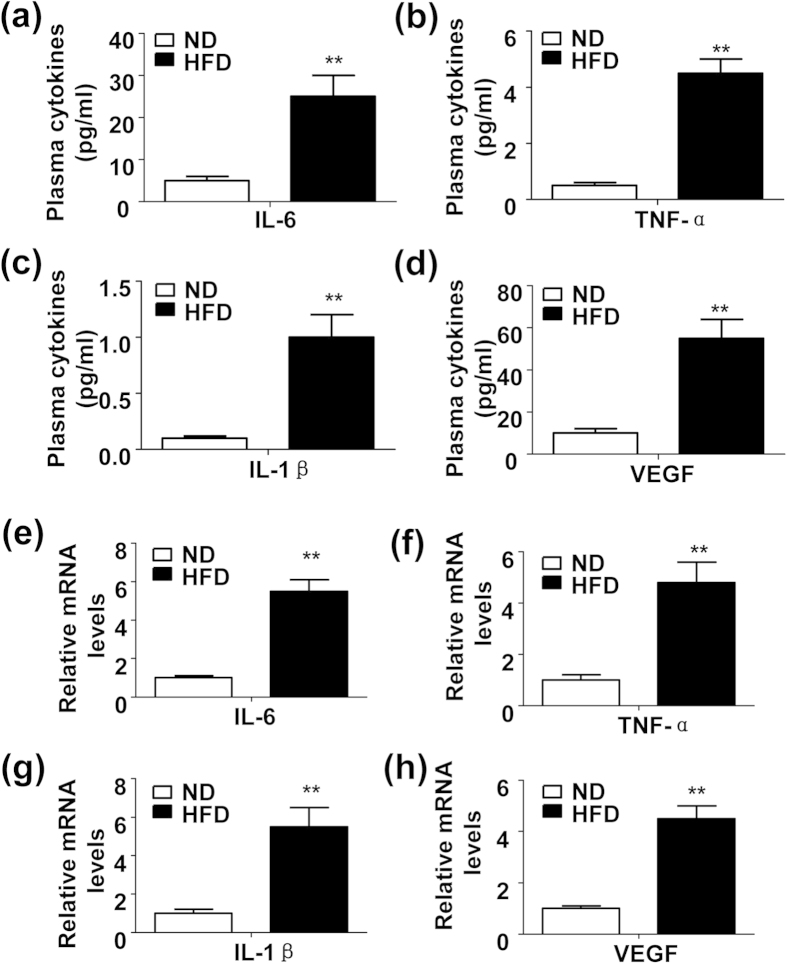
Increased production of proinflammatory cytokines and VEGF in the plasma and chondrocytes in high fat diet (HFD)-feeding mice. (**a**~**d**) levels of plasma IL-6 (**a**), TNF-α (**b**), IL-1β (**c**) and VEGF (**d**) in the C57BL/6 male mice fed with a normal diet (ND) or HFD for 8 weeks. (n = 5, **P < 0.01). (**e**~**h**) Relativ**e** mRNA expression of IL-6 (**e**), TNF-α (**f**), IL-1β (**g**) and VEGF (**h**) in the c**h**ondrocytes isolated from the 8-week ND or HFD-feeding mice. (n = 5, **P < 0.01).

To verify the positive correlation between obesity and OA, we set up an obese mouse model by feeding the mice with a HFD for 8 weeks. Consistent with previous studies^5^,^6^,^18^, HFD induced a low grade inflammation state, characterized by elevated production of IL-6 (Fig. 1a), TNF-α (Fig. 1b) and IL-1β (Fig. 1c) in plasma. Meanwhile, plasma VEGF, a well-documented risk factor of OA, was also induced by HFD (Fig. 1d). Further, the expression pattern of chondrocyte cytokines was identical with the aforementioned ones in plasma (Fig. 1e~h). These results indicated that the HFD induced a low grade inflammation in the mouse cartilages. However, the 8 weeks of HFD-feeding mice didn’t display any signs of osteoarthritis in histomorphology (see Supplementary Fig. S1).

### HFD-induced plasma stearic acid and lactate stimulates proinflammatory cytokine and VEGF production in primary chondrocytes

**Figure 2:**
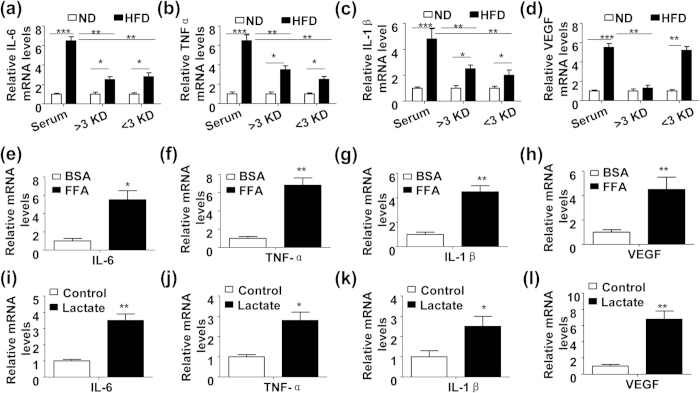
HFD-induced plasma saturated free fatty acid (FFA) and lactate trigger production of proinflammatory cytokines and VEGF in mouse chondrocytes. (**a**~**d**) Relative mRNA levels of IL-6 (**a**), TNF-α (**b**), IL-1β (**c**) and VEGF (**d**) in the mouse primary chondrocytes treated with full or fractionated (>3 KD or <3 KD) serum from the 8-week ND or HFD-feeding mice. (n = 3, *P < 0.05, **P < 0.01 and ***P < 0.005). (**e**~**h**) Relative mRNA levels of IL-6 (**e**), TNF-α (**f**), IL-1β (**g**) and VEGF (**h**) in the mouse primary chondrocytes treated with BSA (5%) or BSA-linked FFA (18:0, 200 μM) for 24 h. (n = 3, *P < 0.05 and **P < 0.01). (**i**~**l**) Relative mRNA expression of IL-6 (**i**), TNF-α (**j**), IL-1β (**k**) and VEGF (**l**) in the mouse primary chondrocytes treated with lactate (25 mM) or vehicle control for 24 h. (n = 3, *P < 0.05 and **P < 0.01).

To explore whether the serum-derived factors are contributors of the chondrocyte inflammation, we collected the serum from the mice with a normal diet (ND) or HFD for 8 weeks. The full serum was then separated into a protein fraction (>3 KD) and a metabolite fraction (<3 KD) to treat the primary chondrocytes from the ND-feeding mice. Comparing to the full serum, protein fraction and metabolite fraction from the ND group, the ones from HFD group notably stimulated mRNA expression of IL-6 (Fig. 2a), TNF-α (Fig. 2b) and IL-1β (Fig. 2c). Unexpectedly, the mRNA expression of VEGF was induced by the HFD group-derived full serum and metabolite fraction but not by the protein fraction (Fig. 2d). While it has been proven that proinflammatory cytokines (TNF-α, IL-1β, etc) are present at high levels in the protein fraction of serum from the HFD-feeding mice^5^,^6^, the proinflammatory molecules in the metabolite fraction of the serum are still not well-known. We performed metabonomics analysis of the metabolite fraction with GC-TOF-MS (see Supplementary Table S1), and verified that at least two elevated metabolic molecules, stearic acid (a kind of saturated FFAs) and lactate in obesity, were functional stimulators of proinflammatory cytokine and VEGF production in primary chondrocytes (Fig. 2e~l). However, the molecular mechanism linking stearic acid and lactate to the cytokine production in chondrocytes will be further explored.

### Stearic acid and lactate induced inflammation response by enhancing protein stability and transcription activity of HIF1α in chondrocytes

**Figure 3:**
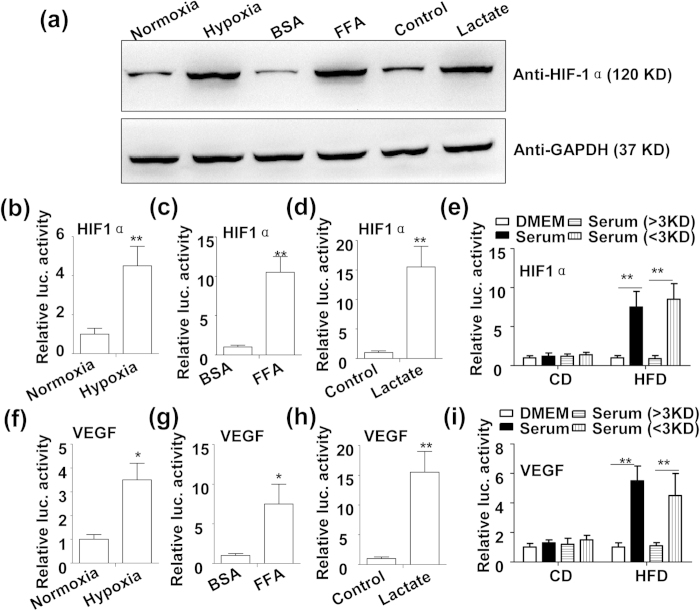
FFA and lactate potentiate HIF1α protein activity in mouse chondrocytes. (**a**) Immunoblotting assay of HIF1α in mouse primary chondrocytes treated with normal oxygen (Normoxia, 20% O_2_), low oxygen (hypoxia, 0.1% O_2_), BSA (5%), FFA (200 μM), lactate (25 mM) and vehicle control for 24 h. (**b**~**d**) Protein stability of HIF1α in response to the treatment with hypoxia. (**b**), 200 μM FFA (**c**) and 25 mM lactate (**d**) for 24 h (n = 4, **P < 0.01). (**e**) Protein stability of HIF1α in response to the treatment with full or fractionated (>3 KD or <3 KD) serum from the 8-week ND or HFD-feeding mice. (n = 3, **P < 0.01). (**f**~**h**) Transcriptional activity of VEGF promoter in response to the treatment with hypoxia (f), 200 μM FFA (**g**) and 25 mM lactate (**h**) for 24 h (n = 3, *P < 0.05 and **P < 0.01). (**i**) Transcriptional activity of VEGF promoter in response to the treatment with full or fractionated (>3 KD or <3 KD) serum from the 8-week ND or HFD-feeding mice. (n = 3, **P < 0.01). Relative luc. activity: relative luciferase activity.

It’s well established that lactic acid is largely produced in hypoxia state, which increases stability and activity of HIF1α, a recently characterized enhancer of chronic inflammation in adipose tissue in obese mice^17^,^19^. Specially, stearic acid and lactate-induced VEGF is a well-known transcriptional target of HIF1α^20^. Thus, we presumed that stearic acid and lactate might regulate the protein stability and transcriptional activity of HIF1α. As expected, similar to hypoxia, both stearic acid and lactate induced protein levels (Fig. 3a), protein stability (Fig. 3b~d) and transcription activity (Fig. 3f~h) of HIF1α in primary chondrocytes. Furthermore, we confirmed that metabolite fraction of serum from the HFD-feeding mice notably induced HIF1α protein stability (Fig. 3e) and transcription activity (Fig. 3i).

**Figure 4:**
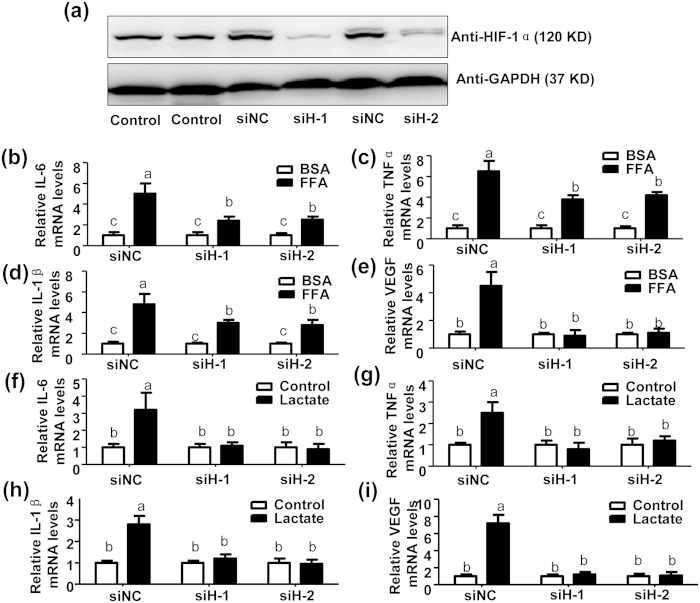
FFA and lactate enhance production of proinflammatory cytokines and VEGF via HIF1α in mouse chondrocytes. (**a**) Immunoblotting assay of HIF1α in primary chondrocytes transfected with a scramble siRNA (siNC, 20 nM), a mouse HIF1α specific siRNA1 (siH-1, 20 nM) or siRNA2 (siH-2, 20 nM) for 36 h. (**b**~**e**) Relative mRNA levels of IL-6 (**b**), TNF-α (c), IL-1β (**d**) and VEGF (**e**) in the mouse primary chondrocytes transfected with siNC (20 nM), siH-1 (20 nM) or siH-2 (20 nM)for 12 h plus additional treatment with BSA (5%) or FFA (200 μM) for 24 h. Values not sharing a common superscript letter differ significantly. (n = 3, P < 0.05). (**f**~**i**) Relative mRNA levels of IL-6 (**f**), TNF-α (**g**), IL-1β (**h**) and VEGF (**i**) in the mouse primary chondrocytes transfected with siNC (20 nM), siH-1 (20 nM) or siH-2 (20 nM) for 12 h plus additional treatment with lactate (25 mM) or vehicle control for 24 h. Values not sharing a common superscript letter differ significantly. (n = 3, P < 0.05).

To verify whether stearic acid and lactate induced cytokine production through HIF1α, mouse HIF1α specific siRNAs were employed to silence the expression of HIF1α in primary mouse chondrocytes (Fig. 4a). As expected, stearic acid-induced production of IL-6 (Fig. 4b), TNF-α (Fig. 4c) and IL-1β (Fig. 4d) could be partly blocked by HIF1α silence, while stearic acid-stimulated VEGF expression was fully prevented by HIF1α deletion (Fig. 4e). In contrast, lactate-induced expression of IL-6 (Fig. 4f), TNF-α (Fig. 4g), IL-1β (Fig. 4h) and VEGF (Fig. 4i) could be fully diminished by HIF1α silence. Those results indicated that stearic acid mediated inflammation response partly through HIF1α, while lactate induced inflammation response in a HIF1α-dependent manner in chondrocytes.

### Stearic acid potentiates LDH-a-dependent lactate production in chondrocytes

**Figure 5:**
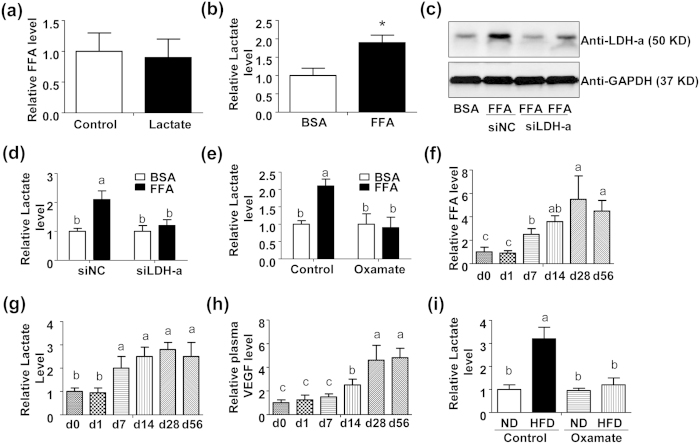
FFA potentiates lactate production via activating LDH-a expression. (**a**) Relative FFA levels in the supernatant of cultured mouse primary chondrocytes treated with lactate (25 mM) or vehicle control. (**b**) Relative lactate levels in the supernatant of cultured mouse primary chondrocytes treated with FFA (200 μM) or 5% BSA. (n = 3, *P < 0.05). (**c**) Immunoblotting assay of LDH-a in mouse primary chondrocytes transfected with siNC (20 nM) or LDH-a specific siRNA (siLDH-a, 20 nM) for 12 h plus additional treatment with BSA (5%) or FFA (200 μM) for 24 h. (**d**) Relative lactate level in the supernatant of cultured mouse primary chondrocytes treated as described in (**c**). (n = 3). (**e**) Relative lactate level in the supernatant of cultured mouse primary chondrocytes treated with BSA (5%) or FFA (200 μM) puls Oxamate (100 nM) or vehicle control for 24 h. Values not sharing a common superscript letter differ significantly. (n = 3). (**f**) Relative plasma FFA levels in the mice with HFD for 0, 1, 7, 14, 28 or 56 days. (n = 5). (**g**) Relative plasma lactate levels in the mice with HFD for 0, 1, 7, 14, 28 or 56 days. (n = 5). (**h**) Relative plasma VEGF level in the mice with HFD for 0, 1, 7, 14, 28 or 56 days. (n = 5). (**i**) Relative plasma lactate levels in the mice fed with HFD for 28 days plus Oxamate (500 mg/kg.d) for one week. (n = 5). From (**d**) to (**i**), values not sharing a common superscript letter differ significantly (P < 0.05).

Given that both stearic acid and lactate could mimic the hypoxia state and regulate HIF1α activity, we presumed that there might be a reciprocal regulatory role between stearic acid and lactate. In primary mouse chondrocytes, lactate treatment exerted no effects on the production of free fatty acids (Fig. 5a), while the treatment with stearic acid notably induced lactate production (Fig. 5b). Further, we demonstrated that stearic acid could induce the expression of LDH-a, a key enzyme for lactate production (Fig. 5c). siRNA-mediated LDH-a silence abolished stearic acid-stimulated lactate production in primary mouse chondrocytes (Fig. 5c~d). To confirm the stimulatory role of stearic acid on lactate production via LDH-a, an inhibitor (Oxamate) of LDH-a activity was employed and identical results were obtained (Fig. 5e and Supplementary Fig. 2). Furthermore, *in vivo* studies were performed to verify the aforementioned findings *in vitro*. The HFD treatment stimulated the levels of free fatty acids (Fig. 5f) and lactate (Fig. 5g) in mouse plasma in a time-dependent manner. Likewise, the plasma VEGF exerted a similar altering pattern as free fatty acids and lactate (Fig. 5h). Interestingly, the HFD-induced lactate production was abolished by additional treatment with the LDH-a inhibitor Oxamate (Fig. 5i).

### Stearic acid stimulates LDH-a-dependent production of proinflammatory cytokines and VEGF

**Figure 6:**
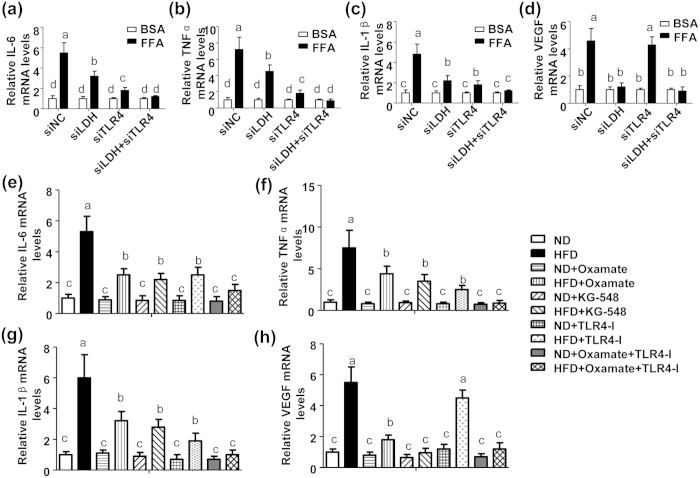
FFA triggers production of proinflammatory cytokine and VEGF partly through LDH-a/HIF1-α pathway. (**a**~**d**) Primary mouse chondrocytes were transfected with siNC (20 nM), siLDH-a (20 nM) or Toll-like receptor 4 specific siRNA (siTLR4, 20 nM) for 12 h, treated with BSA (5%) or FFA (200 μM) for 24 h and then harvested for IL-6 (**a**), TNF-α (**b**), IL-1β (**c**), and VEGF (**d**) mRNA assay with Realtime PCR. (n = 4). (**e**~**h**) Male C57BL/6 mice were fed with a ND or HFD for 8 weeks and treated with LDH-a inhibitor (Oxamate, 500 mg/kg.d), HIF1α inhibitor (KG-548, 1 mg/kg.d), TLR4 inhibitor (TLR4-I, 1 mg/kg.d) or Oxamate (500 mg/kg.d) plus TLR4-I (1 mg/kg.d) for 2 weeks. Then, the cartilage of knee was isolated and subjected for IL-6 (**e**), TNF-α (**f**), IL-1β (**g**), and VEGF (**h**) mRNA assay with Realtime PCR. (n = 5). From (**a**) to (**h**), values not sharing a common superscript letter differ significantly (P < 0.05).

Saturated free fatty acid is the well-documented stimulator of TLR4, mediating inflammation response ubiquitously^5^,^7^,^21^. To identify whether TLR4 is involved in LDH-a/lactate pathway-mediated cytokine production, stearic acid-induced inflammation response in primary chondrocytes with TLR4 or LDH-a silence was observed. Stearic acid-induced mRNA levels of IL-6 (Fig. 6a), TNF-α (Fig. 6b) and IL-1β (Fig. 6c) were largely decreased by a single silence of TLR4 (see Supplementary Fig. S3a) or LDH-a. Combined silence of TLR4 and LDH-a could fully prevent stearic acid-induced proinflammatory cytokine production (Fig. 6a~c). Thus we concluded that stearic acid/LDH-a/lactate pathway was TLR4-independent. Interestingly, stearic acid-stimulated VEGF expression was fully blocked by the silence of LDH-a, but not by TLR4 (Fig. 6d). To verify the aforementioned *in vitro* pathway (stearic acid/LDH-a/cytokines) *in vivo*, we treated the HFD-feeding mice with inhibitors of TLR4 (see Supplementary Fig. S3b), LDH-a or HIF1α (see Supplementary Fig. S3c) and observed cytokine production in mouse cartilage. The HFD-feeding stimulated mRNA expression of IL-6 (Fig. 6e), TNF-α (Fig. 6f) and IL-1β (Fig. 6g), and this effect was partly attenuated by a single treatment of TLR4, LDH-a or HIF1α inhibitors (Fig. 6e~g). Simultaneous administration with TLR4 and LDH-a inhibitors fully prevented HFD-feeding-induced proinflammatory cytokine production in mouse cartilage (Fig. 6e~g). It should be noted that the HFD-feeding-stimulated VEGF expression was prevented by LDH-a or HIF1α inhibitors, but not by TLR4 inhibitor (Fig. 6h). These results verified that stearic acid/LDH-a/HIF1α was a TLR4-independent novel pathway in chondrocytes.

### Positive correlation of plasma lactate, cartilage HIF1α and cytokine levels with the body mass index (BMI) in patients with OA

**Figure 7:**
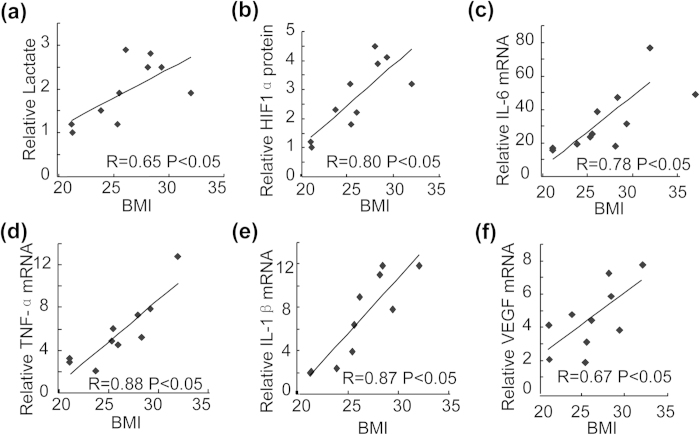
Correlation of plasma lactate, cartilage HIF1α and cytokine levels with the body mass index (BMI) in patients with osteoarthritis. (**a**) Correlation of plasma lactate levels with the BMI in human subjects (n = 10). Pearson’s correlations: R = 0.65, and P < 0.05. (**b**) Correlation of HIF1α protein levels in the cartilages with the BMI in human subjects (n = 10). Pearson’s correlations: R = 0.80, and P < 0.05. (**c**) Correlation of IL-6 mRNA levels in the cartilages with the BMI in human subjects (n = 10). Pearson’s correlations: R = 0.78, and P < 0.05. (**d**) Correlation of TNF-α mRNA levels in the cartilages with the BMI in human subjects (n = 10). Pearson’s correlations: R = 0.88, and P < 0.05. (**e**) Correlation of IL-1β mRNA levels in the cartilages with the BMI in human subjects (n = 10). Pearson’s correlations: R = 0.87, and P < 0.05. (**f**) Correlation of VEGF mRNA levels in the cartilages with the BMI in human subjects (n = 10). Pearson’s correlations: R = 0.67, and P < 0.05.

Aforementioned results revealed that lactate/HIF1α pathway was involved in saturated free fatty acid-induced production of proinflammatory cytokines and VEGF. To further correlate this mechanism to physiological condition, we investigated the levels of plasma lactate, cartilage HIF1α and cytokines in age-matched female human donors with OA. Results revealed that production of plasma lactate (Fig. 7a), cartilage HIF1α (Fig. 7b), IL-6 (Fig. 7c), TNF-α (Fig. 7d), IL-1β (Fig. 7e) and VEGF (Fig. 7f) were positively correlated with the BMI in human donors.

## Discussion

Obesity is causally linked to OA, with the molecular mechanism being not fully understood^2^,^22^. Biomechanics stress (overweight), cytokines (IL-6, TNF-α, IL-1β, VEGF, etc) and hormones (leptin, etc) were identified as key risk factors linking obesity to the setup and progression of OA^9^,^10^. However, the metabolic changes in obesity affecting the cytokine production were still obscure. Here in this study, we are the first to demonstrate that stearic acid stimulated LDH-a-dependent production of lactate, which further stabilized HIF1α protein, and promoted OA-related cytokine expression in chondrocytes. Our work identified a novel pathway (LDH-a/HIF1α/cytokines) mediating saturated free fatty acid-induced cytokine production, independent of TLR4 signaling in chondrocytes.

The levels of saturated free fatty acids (stearic acid, etc) in plasma were increased in obese individuals. Those saturated free fatty acids exerted proinflammatory roles through TLR4 in multiple cells, including monocytes, macrophages, adipocytes and chondrocytes^5^,^23^,^24^,^25^,^26^. In this study, we demonstrated that stearic acid-induced LDH-a/lactate axis was TLR4-independent. However, the precise mechanism linking free fatty acids to LDH-a expression is still not clear. Previous studies revealed that uptake of free fatty acids were increased in multiple cells in obesity^27^,^28^,^29^. We presumed that the chondrocytes might also be overloaded with fatty acids and the over-consumption of oxygen in fatty acid oxidation might generate a hypoxic circumstance, inducing LDH-a-dependent glycolysis and lactate production in chondrocytes.

Lactate was known to stabilize HIF1α protein under normoxia conditions in a latest study^30^, which is consistent with our present work. The role of lactate in inflammation was contradicted in different cells^31^,^32^,^33^. Here we identified the proinflammatory role of lactate via HIF1α in chondrocytes. Interestingly, the production of lactate was tightly potentiated by saturated free fatty acid in obesity. Those findings indicated that lactate might also be a linker between obesity and OA. However, the precise mechanism of lactate stabilizing HIF1α protein needs further to be investigated.

HIFs are pivotal transcriptional factors which are tightly regulated by oxygen concentration^16^. HIF1α and HIF2α regulate different subgroups of genes, although they share some common targets such as VEGF and GLUT1^16^. In arginine metabolism, HIF1α stimulated iNOS production, while HIF2α induced arginase expression^34^,^35^. In fat tissues of obese mice, HIF1α potentiated proinflammatory cytokine production, while HIF2α attenuated chronic inflammation and insulin resistance^19^,^36^. In the present study, we identified that HIF1α, could mediate stearic acid-stimulated inflammatory response in chondrocytes. The specific activation of stearic acid on HIF1α protein is interesting and needs further to be explored.

**Figure 8:**
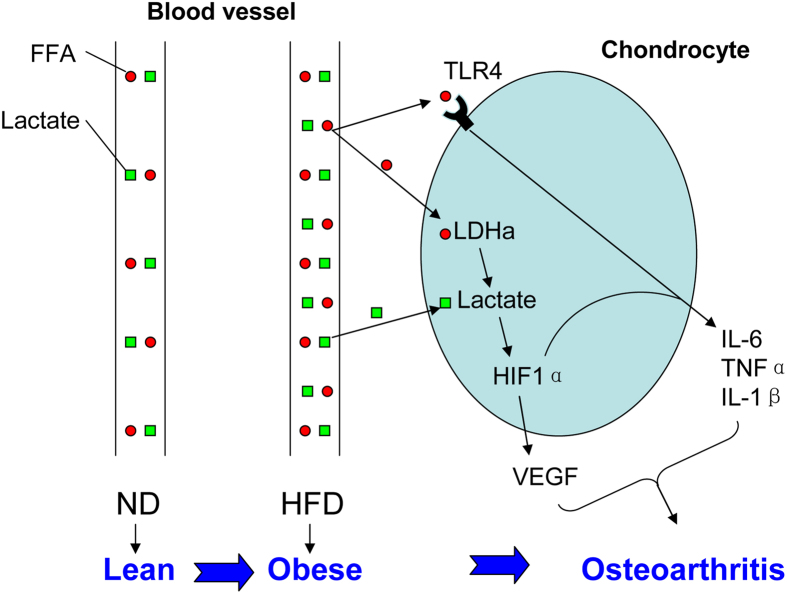
Proposed models for FFA/lactate-mediated HIF1α-cytokine axis in obesity-related osteoarthritis. Briefly, circulatory levels of saturate FFA are increased in diet-induced obesity. Saturated FFA could stimulate proinflammatory cytokine production in chondrocytes directly through TLR4. Meanwhile, the elevated FFA stimulates LDH-a-dependent lactate production in chondrocytes and perhaps also in other cells. Consequently, the levels of circulatory lactate are also increased in response to HFD treatment. The increased lactate would stabilize and activate HIF-1α protein in chondrocytes to stimulate the production of VEGF and proinflammatory cytokines. In summary, saturated FFA could stimulate proinflammatory cytokine production in chondrocytes directly through TLR4 or LDHa-lactate-HIF1α pathway, which provides a potential molecular mechanism linking obesity to chronic inflammation in osteoarthritis.

In all, we identified that elevated circulatory metabolite stearic acid increased lactate levels in the plasma and chondrocytes in a LDH-a-dependent manner. The stearic acid stimulated VEGF and proinflammatory cytokine production through a canonic TLR4 pathway and a novel lactate/HIF1α pathway (Fig. 8). The molecules in both pathways might be potential diagnostic markers and functional therapeutic targets.

## Methods

### Animal studies

All the animal experiments were approved by the Institutional Animal Care and Use Committee at Third Military Medical University (TMMU), and all the experiments were performed in accordance with the “Guide for the care and use of laboratory animals” published by the US National Institutes of Health (publication no. 85–23, revised 1996). All the mice were housed in a pathogen-free facility with a 12-h light, 12-h dark cycle in TMMU. All the mice were provided with food and purified water ad libitum. Each cage contained no more than 5 mice. Six-week-old male C57BL/6 mice were fed with either a normal diet (ND) provided by TMMU (The ingredient is identical with the Research Diets D12450B, 10% calories from fat, Research Diets Inc., New Brunswick, NJ) or a high fat diet (HFD, D12492, fat content 60% by calorie, Research diets, Inc.). The 8-week-ND or HFD-feeding male C57BL/6 mice were treated with LDH-a inhibitor oxamate (#O2751, Sigma, 500 mg/kg.d), HIF1α inhibitor KG-548 (#SML0619, Sigma, 1 mg/kg.d) or Toll-like receptor 4 inhibitor (TLR4-I) (#SML0832, Sigma, 1 mg/kg.d) for 14 days (from the seventh week of HFD) via intraperitoneal injection. We did not see any noticeable reaction or adverse response in the procedure of IP injection.

### Isolation and treatment of primary mouse or human chondrocytes

All the experiments involving human subjects were approved by the ethics committee in TMMU and the informed consent was obtained from all subjects. The methods for isolation and treatment of primary chondrocytes were carried out in accordance with the approved guidelines. Human articular cartilage samples were obtained from the knee joints of female patients (Age = 65 ± 5.6 years, n = 10) undergoing total knee replacement surgery. Primary mouse or human articular chondrocytes were isolated from knee joints and cultured according to the protocol as described in previous study^37^. In the following experiments, cells were treated with different reagents: oxamate (100 nM), KG-548 (500 μM), TLR4-I (100 ng/ml), 5% BSA (#A6003, Sigma) and 200 μM stearic acid (#S4751, Sigma). For hypoxic experiments, the chondrocytes were incubated in an Autoflow NU-8500 incubator (0.1% O_2_ and 5% CO_2_).

### Preparation of saturated free fatty acid

The stock solution of BSA and stearic acid were prepared as described in previous study^7^.

### Enzyme-linked immunosorbent assay (ELISA)

To measure the cytokine levels between lean and obese mice, plasma samples were collected from the C57BL/6 male mice fed with the ND or HFD for 8 weeks. In addition, to test the role of free fatty acid on VEGF production, the 6-week-old male C57BL/6 mice were fed with the HFD. The plasma was collected via tail vein on day 0, 1, 7, 14, 28 and 56. Plasma cytokine levels were measured with TNF-α (#MTA00B), IL-1β (#MLB00C), IL-6 (#M6000B) and VEGF (#MMV00) ELISA Kits from R&D system according to manufacture’s protocols.

### Reverse transcription and realtime PCR

Reverse transcription and realtime PCR were performed as described in the previous report^38^. Briefly, the total RNA was extracted by Trizol reagent (Invitrogen) according to the manufacture’s protocol. RNAs were transcribed into cDNAs using Omniscript (Qiagen, Hilden, Germany). Quantitative Real-Time PCR was performed using the 7900HT Fast Real-Time PCR system (Applied Biosystems, Darmstadt, Germany). The mRNA expression levels were normalized to β-actin. Reactions were done in duplicate using Applied Biosystems Taqman Gene Expression Assays and Universal PCR Master Mix. The relative expression was calculated by the 2(^-DDCt^) method. All the primers used for PCR are available upon request.

### Western blot analysis

Proteins were extracted with RIPA Lysis Buffer and quantified by the BCA kit (Roche, USA). The immunoblotting assay was performed as described previously^6^. Briefly, protein samples were separated by 8 (for HIF1α assay) or 15% (for LDHa assay) SDS-PAGE and transfered to a polyvinylidene difluoride membrane. The membrane was blocked with 5% nonfat milk and incubated with primary antibodies (1:1000) for 10 h at 4 C. The membranes were rinsed 5 times with PBS containing 0.1% Tween 20 and incubated for 1 h with the appropriate horseradish peroxidase-conjugated secondary antibody at 37 C. Membranes were extensively washed with PBS containing 0.1% Tween 20 and incubated with Enhanced Chemiluminescence Substrate (#NEL105001EA, PerkinElmer) for 1 min and the signals were captured using a Bio-Rad ChemiDoc MP System (170–8280). The primary antibodies include Anti-GAPDH (#2118, Cell signaling), Anti-HIF1α (#NB100-449, Novus Biological) and Anti-LDHa (#3558, Cell signaling).

### Loss-of-function studies

HIF1α silence in mouse chondrocytes were performed by transiently transfecting the cells with mouse HIF1α specific siRNAs (siH-1, 5′-GUCACCACAGGACAGUACATT/UGUACUGUCCUGUGGUGACTT-3′ and siH-2, 5′-GCCGCUCAAUUUAUGAAUATT/UAUUCAUAAAUUGAGCGGCTT-3′) or a scramble siRNA as negative control (siNC, 5′-UAGCGACUAAACACAUCAATT/UUGAUGUGUUUAGUCGCUATT-3′). Knockdown of LDH-a and TLR4 expression was performed by target specific siRNAs, siLDHa (sc-45898, Santa Cruz) and siTLR4 (sc-40261, Santa Cruz) respectively. siRNA transfections were carried out using Lipofectanmine^TM^ RNAi Max (Invitrogene) according to manufacture’s instructions.

### HIF1α protein stability assay

A well-constructed reporter gene ODD-Luciferase-pcDNA3 (#18965, Addgene) was employed to detect the stability of HIF1α protein as described previously^30^,^39^. Briefly, the primary mouse chondrocytes were plated in the 96-well-plate (10^5^ cells in each well) and transiently transfected with the reporter construct (working concentration: 0.4 μg/ml). Then, the cells were treated with hypoxia (0.1% O_2_), FFA (200 μM) or Lactate (25 mM) for 24 h. Finally, the luciferase activity was positively correlated with HIF1α protein stability.

### Measurement of HIF1α transcriptional activity

VEGF is a well-known target gene of HIF1α^20^. A 3050-bp (−3000/+50) DNA fragment harboring the mouse VEGF promoter was cloned into pGL4-Basic vector. After transfection with this construct, the luciferase activity indirectly indicates the transcriptional activity of HIF1α.

### Lactate and free fatty acid assay

The supernatant of cultured mouse primary chondrocytes treated with FFA (200 μM) or 5% BSA for 24 h was collected. The 6-week-old male C57BL/6 mice were fed with the HFD. The plasma was collected via tail vein on day 0, 1, 7, 14, 28 and 56. In addition, the plasma from a series of patients with osteoarthritis was also collected. The lactate and the free fatty acid levels in those collected plasma and supernatant were measured using the Lactate Assay Kit (BioVision, USA) and Free Fatty Acid Quantification Kit (K612-100, BioVision, USA) according to the manufacture’s instructions, respectively.

### LDH activity test

LDH Activity Assay Kit (#K726-500, Biovision, Tucson, AZ, USA) was used to determine the intracellular LDH activity. In this test, LDH reduces NAD to NADH, which interacts with a specific probe to produce a color (λ_max_ = 450 nm), which is then detected by colorimetric assay. Results were expressed as percentage of LDH activity normalized to protein concentration, which were measured by BCA protein assay kit (Roche, USA).

### Statistical analysis

All data are expressed as mean ± S.E.M. and were analyzed by either one-way ANOVA or two-tailed unpaired Student’s t test. For each parameter of all data, *P < 0.05, **P < 0.01, ***P < 0.005 and values not sharing a common superscript letter differ significantly (P < 0.05).

## Additional Information

**How to cite this article**: Miao, H. *et al.* Stearic acid induces proinflammatory cytokine production partly through activation of lactate-HIF1α pathway in chondrocytes. *Sci. Rep.* **5**, 13092; doi: 10.1038/srep13092 (2015).

## Supplementary Material

Supplementary Information
