# Supplementary material for: Stearic acid induces proinflammatory cytokine production partly through activation of lactate-HIF1α pathway in chondrocytes
Source: Sci Rep. 2015 Aug 14;5:13092. doi: 10.1038/srep13092 (PMC4536527; doi:10.1038/srep13092)
Supplement: Supplementary Information [file srep13092-s1.pdf]

Stearic acid induces proinflammatory cytokine production partly through activation of lactate-HIF1α pathway in chondrocytes

Hongming Miao[1#*], Liang Chen[2#], Lijun Hao[1], Xuan Zhang[1], Yujuan Chen[1], Zhihua Ruan[1], Houjie Liang[1*]

[1]Department of Oncology, Southwest Hospital, Third Military Medical University, Chongqing 400038, China

[2]Department of Orthopedics, Daping Hospital, Third Military Medical University, Chongqing 400042, China

***Correspondence:**

Houjie Liang, M.D., Ph.D., Department of Oncology, Southwest Hospital, Third Military Medical University, Chongqing 400038, China; Tel: 86-13808392801; Email: lianghoujie@sina.com

Hongming Miao, M.D., Ph.D., Department of Oncology, Southwest Hospital, Third Military Medical University, Chongqing 400038, China; Tel: 86-13678484686; Email: hongmingmiao@sina.com

#These authors contribute equally to this work.

Supplementary information

Includes Supplementary table S1 and Supplementary FigureS1-S3

Supplementary table

Table S1. Different levels of metabolites in the serum from ND or HFD-feeding mice

The 6-week-old male C57BL/6 mice were fed with a ND or HFD for 8 weeks, and then the serum was collected for the GC-TOF-MS assay. Each group included 4 mice. ID, the serial number of the metabolite in the sample library; Similarity, the similarity between the measured metabolite and the one in sample library (High scores indicate high similarity)

ID	Metabolites	Similarity	MEAN (ND)	MEAN (HFD)	P value	Fold change
34	Lactate	995	0.011823434	0.086423157	0.00156715	7.309480181
122	proline	982	0.523475039	0.362431673	0.040684898	0.692357125
125	glycine	972	0.115995433	0.166322484	0.047596521	1.43387097
351	stearic acid	968	0.035875122	0.1842145	0.00145412	5.134881493
376	sucrose	840	0.002514939	0.010938095	0.006261707	4.34924863
168	Bis(2-hydroxypropyl)amine	540	0.006826595	0.001306435	0.038774408	0.191374324
311	Galactonic acid	534	2.552158337	1.151063374	0.042887057	0.451015659
127	2,3-Dihydroxypyridine	472	0.004679784	0.002736028	0.00141904	0.584648351
224	4-hydroxyphenylacetic acid	356	0.010298379	0.005752932	0.029672316	0.558625003
209	beta-Glutamic acid	352	0.003890995	0.000729298	0.042248024	0.187432263
175	Ethyl cinnamate	328	0.0000137	0.000930405	0.029441225	67.91277372
101	2-ketoadipate	320	0.00626273	0.004108912	0.016830235	0.656089597
247	3,6-Anhydro-D-galactose	277	0.003957523	0.000618044	0.00313576	0.156169402

Supplementary figure legends

Figure S1

Histological analysis of articular cartilage from the ND-or HFD-feeding mice. The 6-week-old male C57BL/6 mice were fed with a ND or HFD for 8 weeks. Then, the knee joints were excised and subjected to H&E staining. Representative images were displayed.

Figure S2

Oxamate inhibits LDH activity in a dose dependent manner. The primary mouse chondrocytes were isolated from the knee joint of 14-week-old male mice on a normal diet. The 12-well-plate was seed with 1 million cells in each well. The cells were treated with Oxamate in different doses (0, 10, 100, 1000 nM) for 24 h. Then, the cells were harvested for LDH activity assay. Values not sharing a common superscript letter differ significantly. (n=3, $P<0.05$)

Figure S3

(a) Chondrocyte TLR4 was effectively silenced by siRNA transfection. The primary mouse chondrocytes from the knee joint of 14-week-old male mice on a normal diet were cultured and transfected with a mouse TLR4 specific siRNA (siTLR4, 20 nmol/ml) or a scramble control siRNA (siNC, 20 nmol/ml) for 36 h. Then, the cells were harvested for the mRNA assay of mouse TLR4. (n=3, **$P<0.01$)

(b) TLR4 inhibitor (TLR4-I) efficiently suppressed LPS-stimulated IL-1β mRNA levels in primary mouse chondrocytes. The primary mouse chondrocytes were treated with PBS control or LPS (100 ng/ml) plus different doses of TLR4-I (0, 10, 100, 1000 ng/ml) for 24 h. Then, the cells were collected for mRNA assay

of mouse IL-1β. The relative levels of IL-1β indicated the signal strength of TLR4 which is specifically stimulated by LPS. Values not sharing a common superscript letter differ significantly. (n=3, $P<0.05$)

(c) HIF1α activity was inhibited by KG-548 in a dose dependent manner. The primary mouse chondrocytes were transfected with a reporter gene containing the mouse VEGF promoter (0.4 μg/ml), and then treated with KG-548 in different doses (0, 5, 50, 500 μM) for 24 h. Then, the luciferase (luc.) activity was measured to indicate the transcription activity of HIF1α. Values not sharing a common superscript letter differ significantly. (n=3, $P<0.05$)

Figure S1

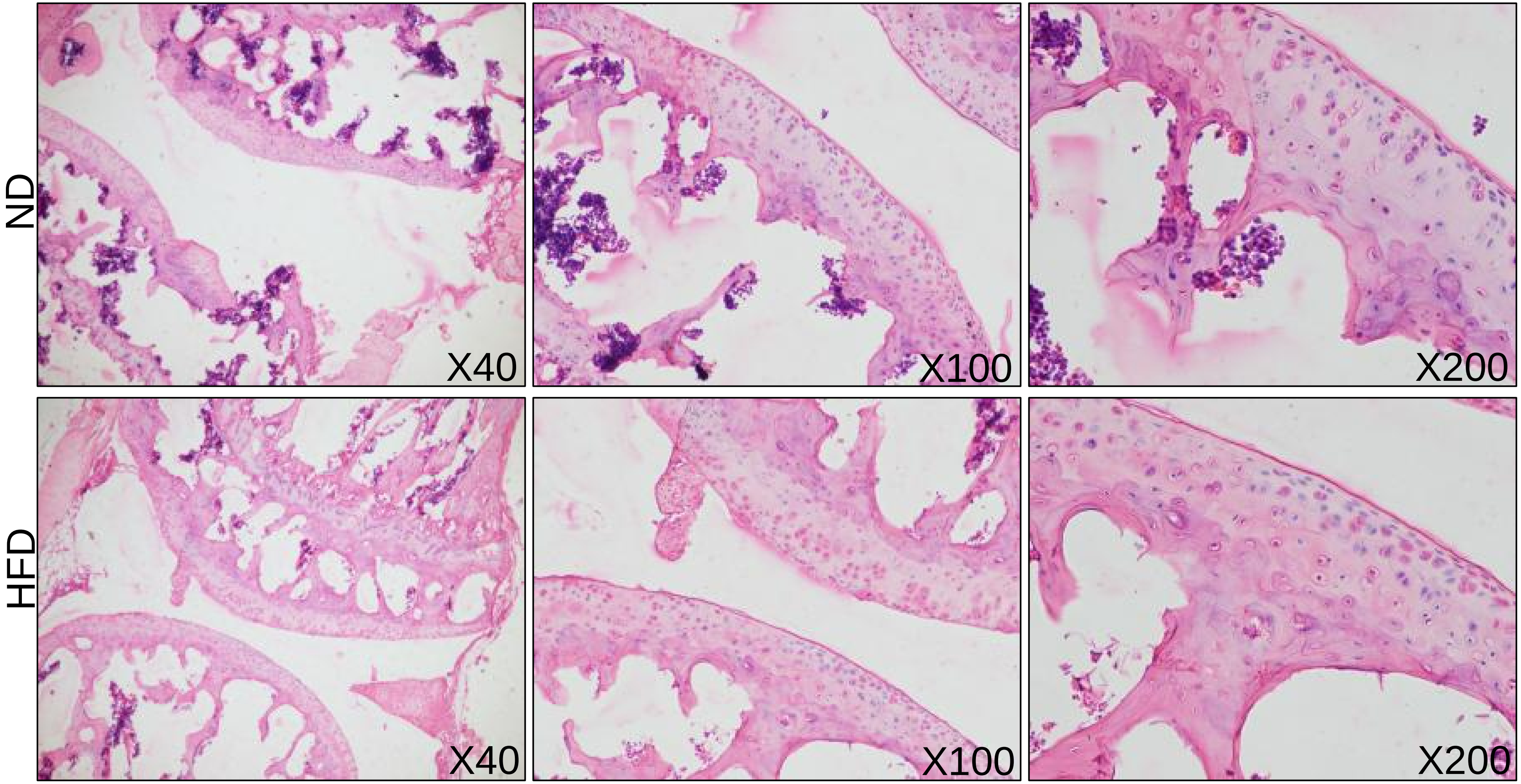

Figure S2

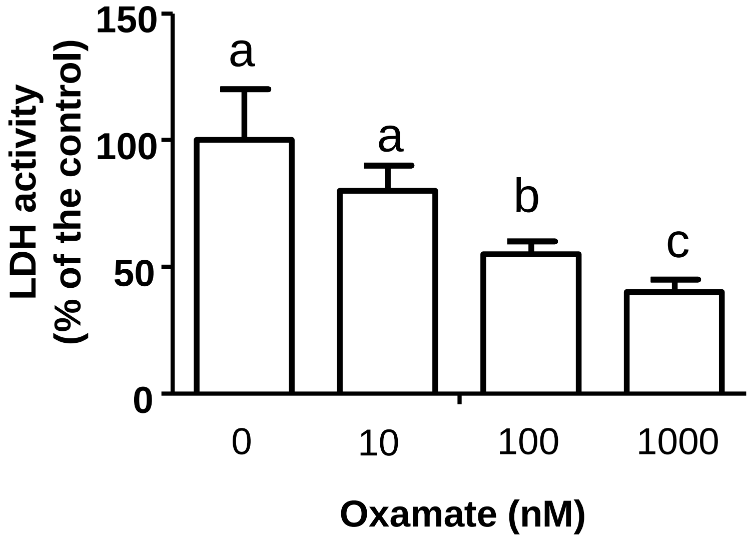
LDH activity
(% of the control)
150
100
50
0
a
a
b
c
0
10
100
1000
Oxamate (nM)

Figure S3

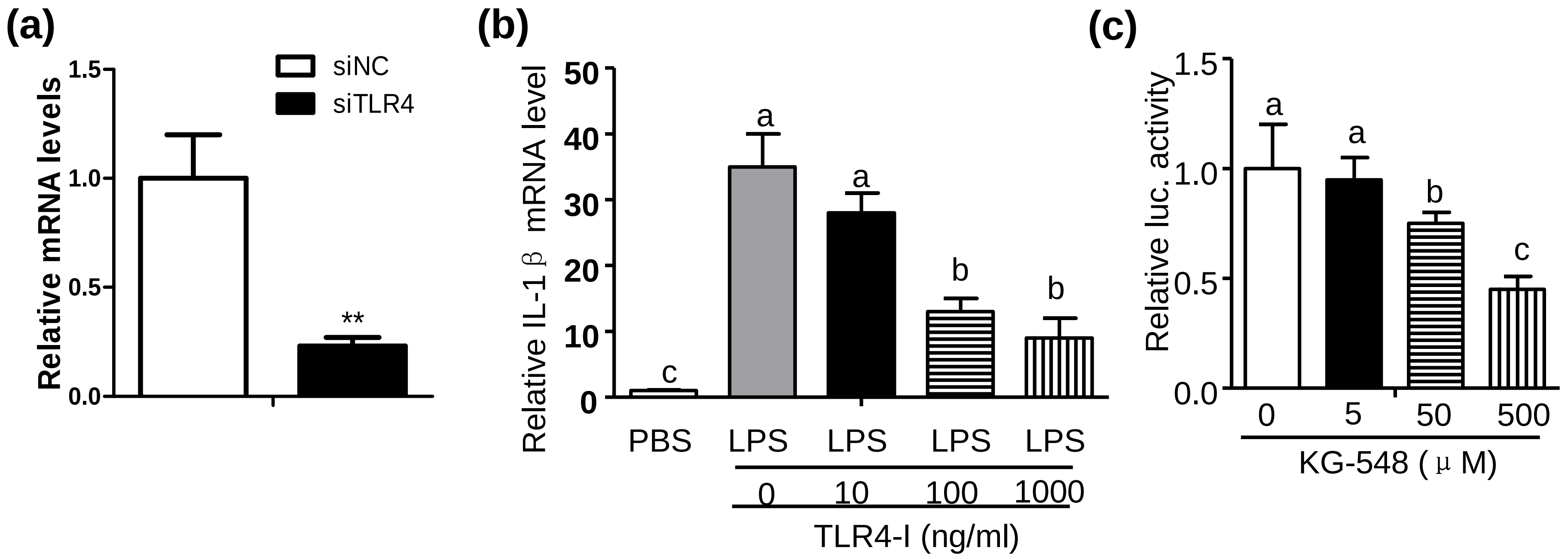
(a)
Relative mRNA levels
1.5
1.0
0.5
0.0
siNC
siTLR4
**
(b)
Relative IL-1β mRNA level
50
40
30
20
10
0
a
a
b
b
c
PBS
LPS
LPS
LPS
LPS
0
10
100
1000
TLR4-I (ng/ml)
(c)
Relative luc. activity
1.5
1.0
0.5
0.0
a
a
b
c
0
5
50
500
KG-548 (μM)
